# Technical steps of open radical cystectomy and orthotopic neobladder to achieve the goals of “minimally invasive surgery”?

**DOI:** 10.4103/0970-1591.70596

**Published:** 2010

**Authors:** Anil Mandhani, Anand Dharaskar, Rakesh Kapoor

**Affiliations:** Department of Urology, Sanjay Gandhi Post Graduate Institute of Medical Sciences, Lucknow, Uttar Pradesh, India

**Keywords:** Open radical cystectomy, minimally invasive surgery, laparoscopic radical cystectomy, robotic radical cystectomy

## Abstract

Technical modifications in open approach to radical cystectomy and orthotopic neobladder (ONB), that is, Pfannenstiel incision, single urethral catheter, internal splint, and extraperitonealization of the ONB were done in 36 patients. Median operative time was 300 (240–360) min. Median time to move the bowel and start of oral intake was 4 days (2–8) days. Major complications occurred in 3 (8.33%) patients. Mean postoperative pain score was 2 (1–4). These modifications in open radical cystectomy resulted in better cosmesis, less pain, and more comfort to the patients as they had to carry one urobag for 3 weeks.

## INTRODUCTION

Conventionally open radical cystectomy and orthotopic neobladder (ONB), which is considered to be a gold-standard treatment for nonmetastatic muscle invasive bladder cancer is done transperitoneally with vertical midline incision extending above the umbilicus.[1,2] Diversion entails suprapubic tube, urethral catheter, 2 ureteric splints, and 1 drainage tube, and patients have to carry 4–5 urobags for 3 weeks.

The minimally invasive surgery (MIS), such as laparoscopic radical cystectomy (LRC) and robotic radial cystectomy (RRC) have been described to achieve less blood loss, less pain, early recuperation, and better cosmesis by avoiding large midline scar.[3–5] Shortcomings of MIS are steep learning curve, operating time, cost of the procedure, and predilection of ileal conduit over the ONB for urinary diversion.[5]

Herein we describe the technical modifications in open radical cystectomy and ONB reconstruction to gain the advantages of MIS.

## TECHNIQUE

### 

#### Modification in Pfannenstiel incision

**Figure 1:**
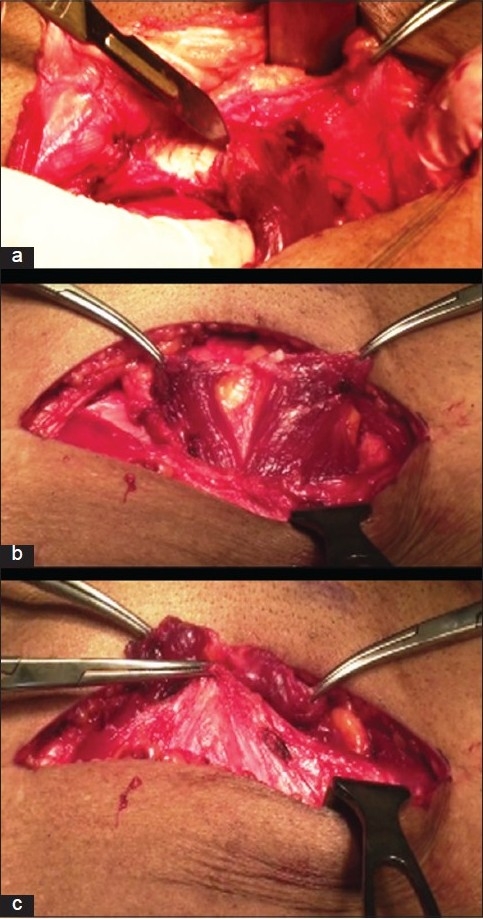
Modification of Pfannenstiel incision

**Figure 2:**
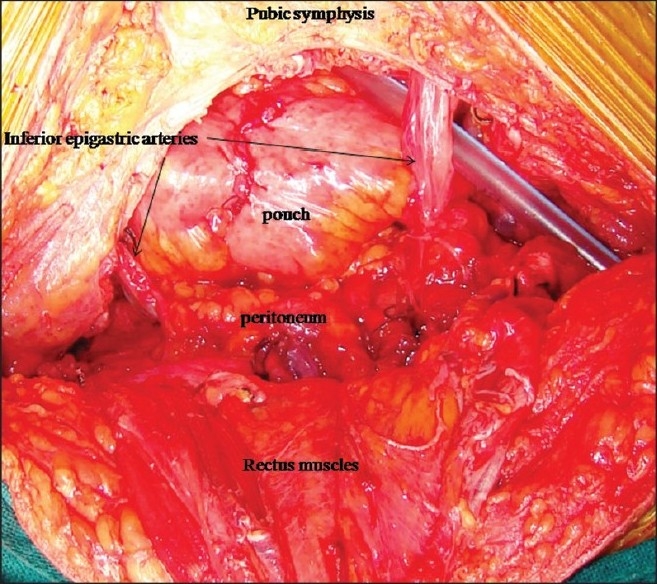
Extraperitonalized neobladder with preserved bilateral inferior epigastric arteries

Pfannenstiel incision was given close to the upper border of the pubic symphysis and was deepened to the rectus sheath. The tendinous insertion of both the rectus muscles was detached using a scalpel leaving the periosteum attached to the muscle [Figure 1a]. The rectus sheath was not separated from their attachments with the rectus muscle as described in Cherney’s modifications of Pfannenstiel incision, that is, detachment of recti. This helped in reducing the strain on rectus tendon to the pubis once the tendon is re-attached and the rectus sheath is sutured [Figures 1b and c]. Both the inferior epigastric arteries were saved [Figure 2].

#### Cystectomy and Neobladder formation

Cystectomy was done extraperitoneally in a retrograde approach. The peritoneum was incised vertically and urachus was detached from the midline and then the cystectomy part was completed. Extended lymphadenectomy up to the bifurcation of aorta was done and lymph nodes were set in separate packets. Sixty centimeters of the small bowel, 12” proximal to the ileocaecal junction was isolated. The distal 40 cm was configured in N shape to make a pouch and proximal 20 cm was left as an afferent loop for ureteric anastomosis as Studer limb. All the bowel anastomoses were hand sewn using 3-0 vicryl.

#### Extraperitonealization of the neobladder

The peritoneum was well separated from the abdominal wall proximally up to the bifurcation of the aorta before opening it for cystectomy. This helped in extraperitonealization of the pouch at the end of the procedure [Figure 2].

#### Testing for watertight suture line of the neobladder

Once the pouch is made, it was filled with 250 cc of saline to check for any leak, which was later reinforced with 3-0 vicryl.

#### Single catheter drainage of neobladder

**Figure 3:**
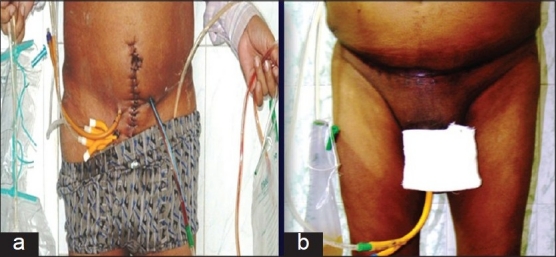
Convenience of carrying 2 bags as compared with 5 bags in patient with conventional radical cystectomy

Conventionally, both the ureteric splints are taken out externally through the abdominal wall along with the 3 additional tubes, that is, suprapubic tube, urethral catheter, and a drain and therefore, the patients have to carry 5 urine bags [Figure 3a]. With these modifications only 2 bags are to be carried by the patient, which makes patients mobility less restricted [Figure 3b].

#### Internal drainage

**Figure 4:**
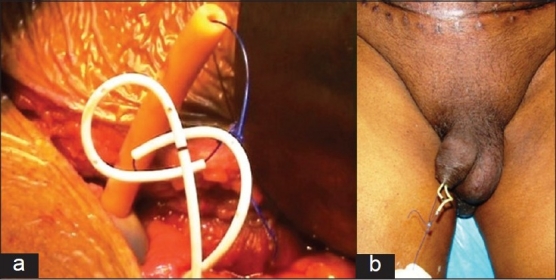
Internal double-J stents attached to the Prolene string and taken out through the lumen of the Foley catheter

Instead of external splints, double-J stents were used internally across the ureteroilial anastomosis. Both the double-J stents were tied with nonabsorbable 0 Prolene and then the Prolene suture was taken out through the inside of urethral catheter [Figure 4a and b]. This arrangement was made with an idea that if Foley catheter was blocked and required to be changed, then the string attached to the double-J stent should still be there in the urethra for its removal at 21 days.

#### Foley catheter

A 2-way Foley catheter of 22 or 24 F with extra eyes made on its tip distal to the balloon was used. Gravity-driven bladder wash was started from postoperative day 2 and frequent milking of the urobag tube was ensured.

#### Outcome measures

All the patients were evaluated for blood loss, operating time, perioperative complication according to the Clavien grades,[6] time to bowel movement (passage of flatus), pain score, and analgesic requirement.

## RESULTS

From October 2007 to February 2010, 36 patients (31 males and 5 females) with a median age of 54.5 (18–73) years underwent operation. The median operating time was 300 (240–360) min. The median time to move the bowel (passage of flatus and presence of bowel sound) was 4 days (2–8) days. The median blood loss was 600 mL (400–1000 mL). Blood transfusion was done in all the patients as mean preoperative hemoglobin was 10.6 gm (9–13.7 gm%). Prolonged urinary leak (more than 5 days) was observed in 3 patients, which stopped on day 10 and 14 in 2 and the third one required bilateral nephrostomy as he had a leak from the ureteroilial anastomosis. Double-J stent could be taken out easily by pulling out the Prolene thread along with the Foley catheter at 3 weeks. No cystogram was performed before removing the Foley catheter. The median hospital stay was 10 (7–20) days.

Pfannenstiel incision did not hinder extended lymphadenectomy. The median number of lymph nodes removed was 14 (7–22).

The median postoperative pain score on visual analog scale was 2 (1–4). The median requirement of analgesics (Tramadol hydrochloride) was 700 mg (400–1000 mg). Patients’ comfort and mobility in postoperative period was better as only one catheter was there for 3 weeks. All the cases had negative surgical margins.

Perioperative complications categorized according to the Clavien grading system have been summarized in Table 1. Three of the 36 patients had prolonged drainage for 12 and 14 days but this leak did not result in any ileus or peritonitis as the whole pouch was extraperitoneal and the urine did not come into contact with the peritoneum. Two patients responded to conservative treatment. One required bilateral nephrostomy as the leak was from the ureteroilial anastomosis. Another patient of Clavien grade 3 complication had a re-exploration for bowel obstruction. One patient died in the postoperative period of severe local wound infection leading to septicemia and multiorgan failure.

**Table 1:** Postoperative complications according to the Clavien grading[6]

Grade	Definition	Number of patients
0	No event observed	0
1	Oral medications or bedside intervention (osteitis pubis)	2
2	Intravenous medications, total parenteral nutrition, enteral nutrition, or blood transfusion	36
3	Interventional radiology, therapeutic endoscopy, intubation, angiography, or operation	2
4	Residual lasting disability requiring major rehabilitation or organ resection	0
5	Death	1

## DISCUSSION

Open radical cystectomy with ONB is the gold standard for treating muscle invasive bladder cancer.[1,2] In a recent review, MIS was suggested for radical cystectomy in the form of LRC and RRC to minimize the postoperative discomfort and give better cosmesis but it needs to be evaluated further for long-term oncologic implications.[5]

According to the data from an international registry on LRC in 572 patients, the mean operating time was 6.2 h with only 53% having ONB. The mean length of hospital stay was 13 days (range 3–90 days). Intraoperative and postoperative complications occurred in 33 (7%) and 139 (28%) patients, respectively.[3] Similarly, in one recent series of RRC, the operating time was reported to be 6.1 h.[4] With a majority of the patients having ileal conduit, the operating time for orthotopic pouch would be much more than the reported mean of 6 h. The median operating time in the present series was 5 h for making an ONB without the use of surgical staplers. The shorter operating time is an important step of minimally invasive surgery and it would reduce the anesthesia time and the need for elective ventilation in postoperative period.

Due to Pfannenstiel incision and detachment of rectus muscle, the whole pelvis was easier to access without a retractor being applied to the lateral edges as in the case of vertical incision. Due to extraperitoneal approach and minimal handling of the intestine, early bowel movement was observed in a majority of the cases. Similar advantage has been reported by adopting extraperitoneal approach with vertical midline incision.[7]

The use of single urethral catheter did not lead to any untoward events. The patients were more comfortable carrying one bag for 3 weeks after the drain was taken out. Suprapubic catheter is not free from morbidity and has been reported to cause persistent neobladder cutaneous fistula.[8,9] With the single urethral catheter there is always an apprehension of the catheter getting blocked. Frequent emptying of the large size and good quality urobag tube has not resulted in any catastrophe. In 2 out of 36 patients there was prolonged drainage from the pouch for 12 and 14 days without any consequence as the ONB was extraperitoneal and whatever urine leak occurred, it remained extraperitoneal.

**Figure 5:**
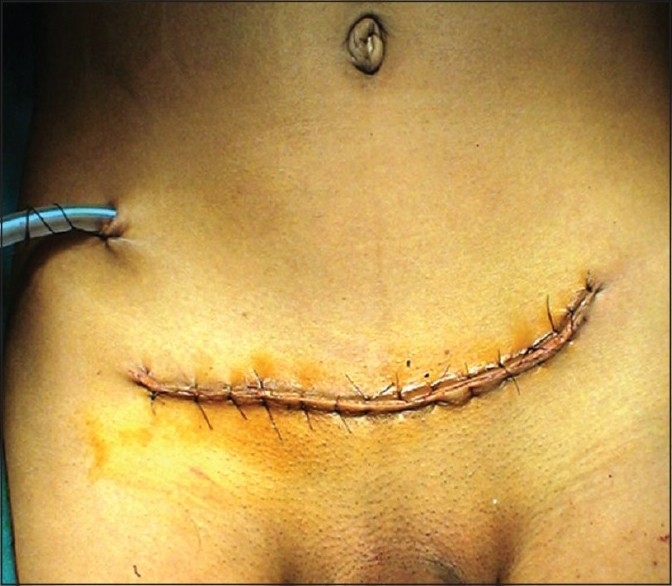
Pfannenstiel incision giving better cosmesis

There has been a stress on describing complications following radical cystectomy using instrument for the outcome measure. In a recent large series of 1142 patients of radical cystectomy, complications of Clavien grade 3–5 occurred in 13%.[10] In the present series complications of grade 3–5 have occurred in 8.33% of our patients [Table 1]. These technical modifications give less postoperative pain, early recuperation, and better cosmesis [Figure 5]. At the early part of our experience when we were using electrocautery to detach the rectus muscle, we had 2 patients who developed osteitis pubis, which responded to conservative treatment. After the modifications described we have not had any case of osteitis pubis [Table 1].

## CONCLUSIONS

Technical modifications in open radical cystectomy and ONB conceptually offer the advantages of minimally invasive surgery, such as less need for analgesics, reduction in length of hospitalization, early recovery, and better cosmesis.
